# Expression and Characterization of a Novel Cold-Adapted and Stable β-Agarase Gene agaW1540 from the Deep-Sea Bacterium *Shewanella* sp. WPAGA9

**DOI:** 10.3390/md19080431

**Published:** 2021-07-29

**Authors:** Wenxin Wang, Jianxin Wang, Ruihua Yan, Runying Zeng, Yaqiang Zuo, Dingquan Wang, Wu Qu

**Affiliations:** 1 Marine Science and Technology College, Zhejiang Ocean University, Zhoushan 316000, China; Wangwenxin5688@163.com (W.W.); jxwang@zjou.edu.cn (J.W.); viento123@126.com (Y.Z.); regen8616@163.com (D.W.); 2 Technical Innovation Center for Utilization of Marine Biological Resources, Ministry of Natural Resources, Xiamen 361000, China; 18013087015@stu.hqu.edu.cn (R.Y.); zeng@tio.org.cn (R.Z.)

**Keywords:** neoagaro-oligosaccharides, β-agarase, cold-adapted, stable, deep-sea

## Abstract

The neoagaro-oligosaccharides, degraded from agarose by agarases, are important natural substances with many bioactivities. In this study, a novel agarase gene, agaW1540, from the genome of a deep-sea bacterium *Shewanella* sp. WPAGA9, was expressed, and the recombinant AgaW1540 (rAgaW1540) displayed the maximum activity under the optimal pH and temperature of 7.0 and 35 °C, respectively. rAgaW1540 retained 85.4% of its maximum activity at 0 °C and retained more than 92% of its maximum activity at the temperature range of 20–40 °C and the pH range of 4.0–9.0, respectively, indicating its extensive working temperature and pH values. The activity of rAgaW1540 was dramatically suppressed by Cu^2+^ and Zn^2+^, whereas Fe^2+^ displayed an intensification of enzymatic activity. The *K_m_* and *V_max_* of rAgaW1540 for agarose degradation were 15.7 mg/mL and 23.4 U/mg, respectively. rAgaW1540 retained 94.7%, 97.9%, and 42.4% of its maximum activity after incubation at 20 °C, 25 °C, and 30 °C for 60 min, respectively. Thin-layer chromatography and ion chromatography analyses verified that rAgaW1540 is an endo-acting β-agarase that degrades agarose into neoagarotetraose and neoagarohexaose as the main products. The wide variety of working conditions and stable activity at room temperatures make rAgaW1540an appropriate bio-tool for further industrial production of neoagaro-oligosaccharides.

## 1. Introduction

Agar is the main glycan component that is segregated from the cell wall of red algae, for instance, *Gracilaria* sp. and *Gelidium* sp. [1,2]. As the major biopolymer and supporting structure in seaweed, a cocktail of agarose and agaropectin constitute the molecular structure of agar [3,4]. Agarose, as the main component of agar, possesses a linear structure that is composed of the alternant subunit of β-d-galactose and 3,6-anhydro-l-galactose [5].

Agarases that depolymerize agarose into agarooligosaccharides (AOS) or neoagarooligosaccharides (NAOS) have been found in several gram-negative bacteria isolated from submarine sediments, seaweeds, marine organisms, and seawater [2]. Agarases are divided into two types, owing to the different hydrolysis modes, i.e., α-agarase (EC 3.2.1.158), which acts on the α-1,3 glycoside bonds and yields AOSs with 3,6-anhydro-l-galactose at the reducing ends of products, and β-agarase (EC 3.2.1.81), which cleaves the β-1,4 glycoside bonds to generate NAOS with β-d-galactose at the reducing ends of products [5,6].

Compared to α-agarases, β-agarases have a wider distribution in nature [7]. β-agarases belong to glycoside hydrolase (GH) families, including GH 16, GH 39, GH 42, GH 50, GH 86, and GH118 [2,8,9]. Neoagarobiose (NA2), neoagarotetraose (NA4), neoagarohexaose (NA6), neoagarooctaose (NA8), neoagarodecaose (NA10), and neoagarododecaose (NA12) are the usual products of β-agarases [5]. These NAOSs possess multitudinous bioactivities, such as antioxidant, antitumor, anti-obesity, anti-diabetic, anti-inflammatory, anti-cariogenic, skin whitening, and radical-scavenging activities [1,10,11,12], which can be widely used in the fields of food, medicine, and cosmetics.

Until now, many agarases have been thermostable but lacking in activity at low temperatures [5]. Therefore, identifying and sieving out the agarases that possess the ability of hydrolysis at low temperatures is indispensable. However, only limited agarases with cold-adaptation, such as AgaJ5, AgaJ9, and Aga21, have been identified and characterized in recent years. In detail, AgaJ5 retained ~40% of the maximum enzymatic activity at 10 °C [13]. AgaJ9 maintained more than 80% of the maximum activity at 5 °C [8], and Aga21 retained as much as 85% of the maximum activity at 10 °C [14]. However, the agarases that can work at extreme temperatures, such as 0 °C, are rarely reported.

Our former study demonstrated the degradation activities of a deep-sea bacterium *Shewanella* sp. WPAGA9 for three seaweed polysaccharides [15]. In this study, a cold-adapted GH16 β-agarase, AgaW1540, from the genome of *Shewanella* sp. WPAGA9 had been further investigated and characterized. The results showed that the recombinant AgaW1540 (rAgaW1540) can maintain 85.4% of maximum enzymatic activity at 0 °C, indicating an outstanding level of cold-adaptation. The current study provides a potential bio-tool for NAOS production at low temperatures, and an ideal model for researching the cold-adapted mechanisms of agarases.

## 2. Results

### 2.1. Sequence Analysis and Purification of rAgaW1540

**Figure 1:**
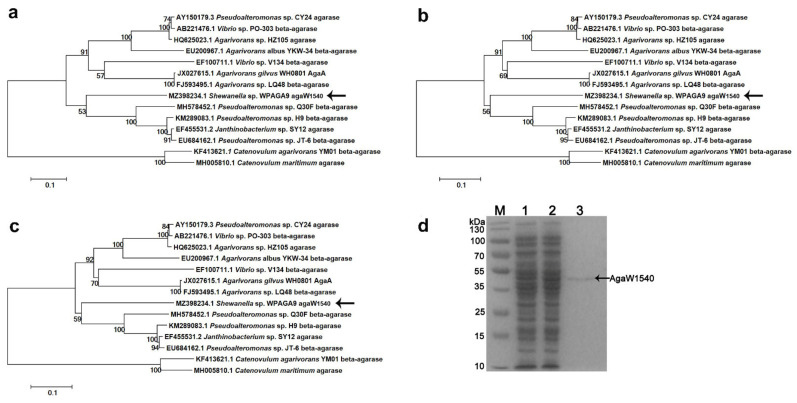
Phylogenetic analysis of the nucleotide sequence of agaW1540 based on the methods of maximum likelihood (**a**), neighbor-joining (**b**), and minimum evolution (**c**) and SDS-PAGE result of purified rAgaW1540 (**d**). The phylogenetic trees were generated by using Mega 5.2 software. Bootstrap values of 1000 replications are displayed at branching points. The scale plate denoting ten nucleotide substitutions per 100 nucleotides is shown at the bottom. The gene of agaW1540 is indicated in bold with arrows. (**d**) SDS-PAGE of the purified rAgaW1540. Lane M, protein maker; Lane 1, cell lysate before induction; Lane 2, cell lysate after induction; Lane 3, purified rAgaW1540. As the figure displayed, the arrow expounds the position of the rAgaW1540 band.

By the annotation against NCBI nr database, agaW1540 (GenBank accession number MZ398234) in the genome of *Shewanella* sp. WPAGA9 was predicted to be a putative β-agarase gene due to its maximum identity of 74.64% with the β-agarase genes from *Agarivorans* sp. HZ105 (GenBank accession number: HQ625023.1) and *Pseudoalteromonas* sp. CY24 (GenBank accession number: AY150179.3). In the phylogenetic trees constructed by the methods of maximum likelihood (Figure 1a), neighbor-joining (Figure 1b), and minimum evolution (Figure 1c), the nucleotide sequence of agaW1540 had the closest phylogenetic relationship with a β-agarase from *Pseudoalteromonas* sp. Q30F (GenBank accession number: MH578452.1). The annotation results from the dbCAN database assigned AgaW1540 into the GH16 family. The gene agaW1540 encoded 453 amino acids with a theoretical molecular weight of 50.96 kDa. AgaW1540 possesses an N-terminal signal peptide of 21 amino acids with a possible cleavage site between position Ala21 and Ala22. The gene agaW1540 was expressed in *E. coli* BL21(DE3) cells using a pEAZY^®^-Blunt E2 expression vector (TransGen Biotech., Beijing, China). The recombinant protein was released by ultrasonication, and was purified by Ni-nitrilotriacetic acid (NTA) Sefinose™ Resin (Tiangen Biotech, Beijing, China). A single distinct band of rAgaW1540 was displayed in sodium dodecyl sulfate-polyacrylamide gel electrophoresis (SDS-PAGE) with a molecular weight of ~50 kDa (Figure 1d), which was consistent with the theoretical molecular weight.

### 2.2. Biochemical Characterization and Enzymatic Features of rAgaW1540

**Figure 2:**
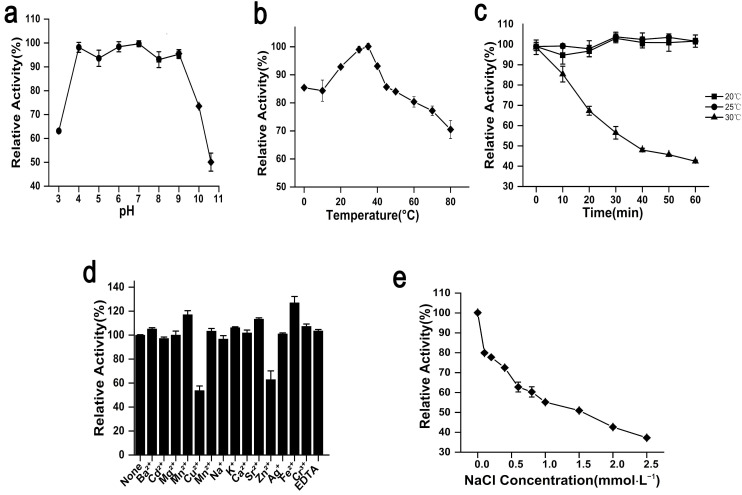
Biochemical properties of rAgaW1540. (**a**) pH effects on the enzymatic activity of rAgaW1540 with the range of 3.0–10.6. (**b**) The temperature effects on enzymatic activity with a temperature range of 0 to 80 °C. (**c**) The thermal stability of rAgaW1540. (**d**) The effect of chemical reagents on the enzymatic activity. (**e**) The effect of NaCl on enzymatic activity with a concentration range of 0–2.5 mol·L^−1^. All the plot values displayed above are relative numbers of the maximum activity of rAgaW1540 (presented as 100%) and repeat three times that are denoted with standard deviation (SD).

The optimum pH of rAgaW1540 was 7.0. Meanwhile, rAga1540 exhibited the maximum activity at an extensive pH coverage, and maintained 98.2%, 93.6%, and 98.4% of the maximum activity at pH 4.0, 5.0, and 6.0, respectively (Figure 2a). The optimum temperature of rAgaW1540 was 35 °C (Figure 2b). rAgaW1540 retained 92.8%, 98.9%, and 93.1% of its maximum activity at 20, 30, and 40 °C, respectively. Furthermore, rAgaW1540 maintained 85.4% of its maximum activity at 0 °C, elucidating that rAgaW1540 is a cold-adapted agarase. 

### 2.3. Stability of rAgaW1540 against Temperatures and Other Potential Inhibitors

rAgaW1540 showed stability at ambient temperatures. The results of thermal stability experiments elaborated that rAgaW1540 maintained 94.7% and 97.9% of its maximum activity after the pre-incubation at 20 °C and 25 °C for 1 h, respectively (Figure 2c). However, the activity of rAgaW1540 was mostly lost after incubation at 30 °C. Among the metal ions additives, Cu^2+^ and Zn^2+^ had negative effects on rAgaW1540; meanwhile, Ba^2+^, Mn^2+^, Ni^2+^, K^+^, Ga^2+^, Sr^2+^, Ag^+^, Cr^2+^, and EDTA slightly promoted the enzymatic activity of eAgaW1540. Fe^2+^ exhibited a promotion that increased the relative activity of rAgaW1540 up to 126.99% (Figure 2d). NaCl exhibited a suppression on the enzymatic activity of rAgaW1540 (Figure 2e), and the *V_max_* and *K_m_* of rAgaW1540 for agarose were 23.4 U/mg and 15.7 mg/mL, respectively.

### 2.4. The Degradation Products of rAgaW1540 for Agarose

**Figure 3:**
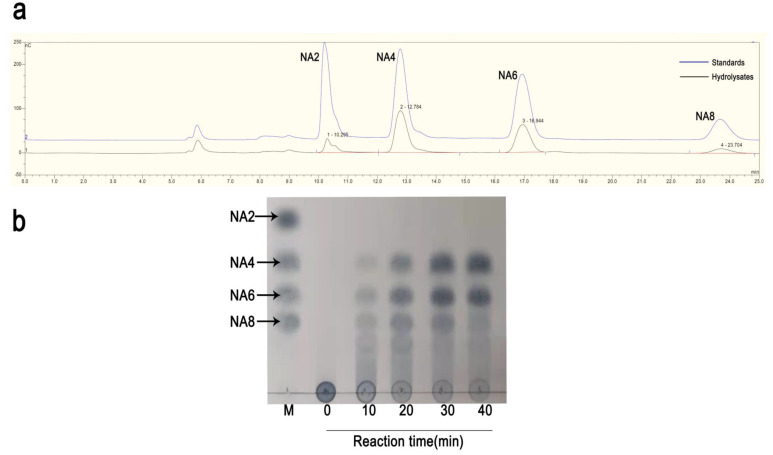
IC chromatogram (**a**) and TLC analysis (**b**) of the hydrolysates of AgaW1540. NA2, neoagarobiose; NA4, neoagarotetraose; NA6, neoagarohexaose; NA8, Neoagarooctaose.

Based on the results of thin-layer chromatography (TLC), agarose was firstly degraded into NA12, NA8, NA6, and NA4 after degradation for 10 min, which suggested rAgaW1540 was an endo-type agarase. Subsequently, NA12 and NA8 were further degraded into NA4 and NA6. The main products of rAgaW1540 were NA4 and NA6 after degradation for 24 h (Figure 3a). To further confirm the hydrolysates of rAgaW1540, the products that were degraded for 48 h were identified by ion chromatography (IC; Figure 3b). The results of the IC analysis revealed that the agarolytic products of rAgaW1540 had similar retention times with NA2, NA4, NA6, and NA8. Furthermore, NA4 and NA6 had the largest proportions among these oligosaccharides, indicating that NA4 and NA6 were the main products of rAgaW1540.

### 2.5. Fermentation Optimization of rAgaW1540 Production

**Figure 4:**
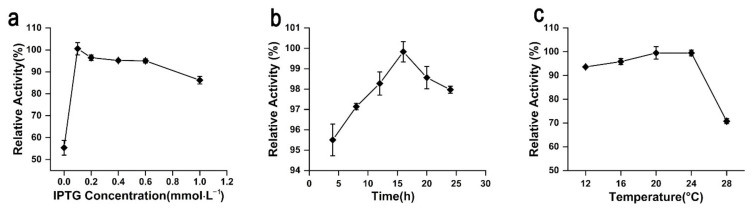
The effects of IPTG concentrations (**a**), reaction time (**b**), and induction temperatures (**c**) on the production of rAgaW1540.

Leakage expression was observed because the activity of rAgaW1540 was detected without the induction of isopropyl-β-d-thiogalactopyranoside (IPTG; Figure 4a). The production of rAgaW1540 peaked at an IPTG concentration of 0.1 mmol·L^−1^ (Figure 4a). High IPTG concentrations could inhibit the production of soluble rAgaW1540 (Figure 4a). The optimum time for inducing the production of rAgaW1540 by IPTG was 16 h (Figure 4b). The production of rAgaW1540 maximized at the induction temperatures of 20–24 °C (Figure 4c).

## 3. Discussion

Herein, we overexpressed a novel β-agarase gene agaW1540 from the genome of a deep-sea bacterium *Shewanella* sp. WPAGA9. The newfound enzyme that belonged to the GH16 family was biochemically characterized as an endo-type β-agarase with the optimal bioactivity at pH 7.0 and 35 °C with the main products of NA4 and NA6. The stability of agarase exerts an important influence on the application of its industrial production [16]. The results indicated that rAgaW1540 was very stable against pre-incubation at ambient temperatures of 20 °C and 25 °C. Until now, most agarases have been difficult to stabilize at room temperature [17], which extremely impeded their industrial utilizations. To conserve their bioactivity, it has been necessary to decrease the storage temperature of the enzymes during the industrial production process, which can cause a waste of energy. Therefore, rAgaW1540, with its outstanding room-temperature stability, could become a less costly agarase alternative.

rAgaW1540 adapted to a broad pH range of 4.0–9.0, as it maintained more than 93% of the maximum activity at these pH values, which was much higher than most of the pH-stable agarases that originated from different marine bacteria [15,17,18]. For instance, Aga3027 has been reported to maintain more than 85% of its enzymatic activity at pH 7.0–9.0 [18]. AgaB is stable at pH 6.5–8.5, as more than 80% of the enzymatic activity was retained [16]. Aga862 retains 80% of its relative activity at pH 3.0–10.0 [19]. The wide pH ranges facilitate the adaptability of rAgaW1540 to pH change during the reaction process. Additionally, the high activity of rAgaW1540 at alkaline pHs could effectively decrease the expenditure of neutralizing alkali in the course of extracting NAOS from the biotransformation of agar, which is a more friendly method of NAOS production [18].

rAgaW1540 had a good cold-adaptation. This agarase maintained 85.42% of its maximum activity at 0 °C. Four β-agarases, including three β-agarases from *Gayadomonas joobiniege* G7 and one from *Pseudoalteromonas* sp. NJ21, have been reported to possess cold-adaptation. These agarases were variously sorted into the GH39, GH42, and GH86 families. In detail, AgaJ5 possesses a prominent cold tolerance, as it retained enzymatic activity of 40% at 10 °C [13]; AgaJ9 sustained 89% of agarase activity at 10 °C [8]; AgaJ10 showed 50% of enzymatic activity at 10 °C [9]; whereas Aga21 retained 85% of enzymatic activity at 10 °C [14]. Nevertheless, the agarases that can work at 0 °C are rarely reported. Our study provides an insight into a novelty agarase with high activity at 0 °C. The cold-adapted rAgaW1540 could maintain efficient activity at low temperatures in extreme conditions, such as deep-sea environments. This property enables the deep-sea strain WPAGA9 to rapidly degrade and utilize the carbon source, which is key for its growth and element cycling in a deep-sea environment. Notably, cold-adaptation is not a common characteristic among the existing agarases, even for the agarases isolated from deep-sea bacteria [5,20]. For example, agarase HZ2 [21], AgaP4383, and Aga4436 [6] from deep-sea bacteria cannot maintain their activities at temperatures lower than 5 °C. Therefore, this study also provides an ideal but rare structure model for future research on the cold-adapted mechanisms of proteins. 

Neoagaro-oligosaccharides, the products hydrolyzed by agarases, possess multifold physiological bioactivities, including prebiotics, antioxidant, anti-inflammatory, anti-obesity, and skin moisturizing effects [10,11,22,23,24]. In this study, NA4 and NA6 were the main products of agarose degradation by rAgaW1540. To date, NA4 has been found to attenuate the inflammatory responses [25]. Additionally, a recent report demonstrated that NA4 can selectively modulate gut microbial community composition and regulate multiple intestine metabolites to alleviate inflammation [26]. NA6 was reported to possess a potential antitumor immunity and act as a cell modulator towards melanoma, and afterwards was verified to have an antivirus inborn immunity against norovirus [12,27]. Owing to these properties, in this study, rAgaW1540 can be further applied in food, medicine, and cosmetic industries.

In summary, this study explores an agarase rAgaW1540 from a deep-sea bacterium. The wide pH and temperature working range make rAgaW1540 stable and adaptable to environmental variation during the reaction process. rAgaW1540 is convenient for storage and transportation due to its strong stability at room temperatures. Notably, rAgaW1540, with its cold-adaptation, could possibly facilitate the carbon cycling in the low temperatures of deep-sea environments and provide a potentially valuable model for researching the mechanisms of protein cold tolerance. 

## 4. Materials and Methods

### 4.1. Sequence Analysis and Cloning of agaW1540

The nucleotide sequence of agaW1540 had been deposited in the GenBank database under the accession number MZ398234. The sequence of agaW1540 was annotated against NCBI nr (ftp://ftp.ncbi.nlm.nih.gov/blast/db, accessed on 7 July 2020) and dbCAN (http://csbl.bmb.uga.edu/dbCAN/, accessed on 7 July 2020) databases. Based on the nucleotide sequences, the agarase genes were aligned by using ClustalW and the phylogenetic analysis was performed by the methods of neighbor-joining, maximum likelihood, and minimum evolution in MEGA 5.2 software (https://www.megasoftware.net/, accessed on 19 July 2021) with a bootstrap value of 1000. The prediction of signal peptide was performed by using SignalP-5.0 (http://www.cbs.dtu.dk/services/SignalP/, accessed on 7 July 2020). *Shewanella* sp. WPAGA9 was cultured in marine broth 2216E (5.0 g peptone, 1.0 g yeast extract, 0.01g iron phosphate, and 1 L seawater; pH 7.4) at 30 °C for 12 h. The genomic DNA of *Shewanella* sp. WPAGA9 (GenBank accession number: CP062058) was extracted by using a TIANamp Bacteria DNA Kit (Tiangen Biotech., Beijing, China). The gene agaW1540 was amplified through PCR with a forward primer (5′-AAACCTAGTATGAATGTTATCTATGCTACTAC-3′) and a reverse primer (5′-CTCTACTTTAAATTGTTGATTGGTTCCG-3′). PCR was conducted using a C1000™ Thermal Cycler (Bio-Rad CO., Ltd., Hercules, CA, USA) and the PCR condition was as follows: 3 min at 95 °C; 25 cycles of 95 °C for 25 s, 55 °C for 25 s, 72 °C for 1 min/kb; and extension at 72 °C for 6 min. The PCR products were purified by Universal DNA Purification Kit (Tiangen Biotech., Beijing, China) and then were ligated with pEAZY^®^-Blunt E2 expression vector (TransGen Biotech., Beijing, China). The recombinant vector harboring agaW1540 gene was transformed into *E. coli* BL21(DE3) pLysS cells.

### 4.2. Expression and Purification of rAgaW1540

The *E. coli* BL21(DE3) pLysS cells harboring the recombinant vector were cultured at 37 °C in LB broth (5.0 g yeast extracts, 10.0 g peptone, 10.0 g NaCl, and 1 L deionized water) including 100 μg/mL ampicillin, and the culture broth was induced by adding 0.1 mM IPTG at 16 °C until the OD_600nm_ reached 0.6–0.8. The cells were collected by centrifugation at 10,000 rpm for 10 min at 4 °C, and then were subsequently resuspended in a Tris-HCl buffer (10 mM; pH 7.4). The resuspended cells were fractured using a sonicator (Scientz, Ningbo, China) at a rate of 3s/2s impulse frequency for 20 min in ice. The Ni-nitrilotriacetic acid (NTA) Sefinose™ Resin (Tiangen Biotech, Beijing, China) was used to further purify rAgaW1540 with 6×His-tagged from the crude enzyme solution. The molecular weight and purity of rAgaW1540 were subsequently estimated by SDS-PAGE and Coomassie brilliant blue staining.

### 4.3. Characterization of Enzymatic Properties

The agarase activity (U) was defined as the amount of the enzyme that released 1 μmol of reducing sugars per minute under certain conditions. In detail, 10 μL enzyme solution was mixed with 240 μL agarose (0.4%; *w*/*v*) with different pHs including 3.0–8.0 (50 mM sodium citrate-dibasic sodium phosphate), 8.0–9.0 (50 mM Tris-HCl), and 9.0–10.6 (50 mM glycine-NaOH), and the mixture was incubated at the temperatures of 0, 10, 20, 30, 35, 40, 45, 50, 60, 70, and 80 °C for 10 min, respectively. Then, 750 μL 3,5-dinitrosalicylic acid (DNS) solution was added, and the resulting sugar was measured by the DNS method [28]. To determine the stability against pH, rAgaW1540 was pre-incubated in the above-mentioned pH buffers at 25 °C for 60 min, and then the residual activity was determined. To determine the thermostability, rAgaW1540 was pre-incubated at 20, 25, and 30 °C for 60 min, and then the residual activity was determined. The effects of metal ions and metal chelators were measured by determining the enzymatic activity of rAgaW1540 in the solution containing Ba^2+^, Cd^2+^, Mg^2+^, Mn^2+^, Cu^2+^, Ni^2+^, Na^+^, K^+^, Ga^2+^, Sr^2+^, Zn^2+^, Ag^+^, Fe^2+^, Cr^2+^, and EDTA at a final concentration of 10 mM. The effect of NaCl concentrations on rAgaW1540 activity was measured by determining the activity at the NaCl concentrations of 0–2.5 mol·L^−1^. The kinetic parameters of rAgaW1540 were measured by determining the activity with the agarose at final concentrations of 0.1–0.6 mg/mL without any metal additive, and the *K_m_* and *V_max_* values were subsequently calculated by the Lineweaver–Burk plot. All determinations were repeated three times.

### 4.4. Identification of the Hydrolysates of rAgaW1540

TLC analysis was conducted to investigate the hydrolysis modes and products of rAgaW1540 in 0, 10, 20, 30, and 40 min. The agarolytic reaction of rAgaW1540 were conducted in a 50 mM Tris-HCl buffer (pH 7.0) with 0.1% agarose (*w*/*v*) at 25 °C. The products were developed on a silica gel 60 plate (Merck, Darmstadt, Germany) with the developing solvent containing n-butanol, acetic acid, and water (2:1:1; *v*/*v*/*v*). The products on the plates were visualized by heating with 20% H_2_SO_4_ at 130 °C for 10 min. To further identify the degradation products, the hydrolysates of rAgaW1540 were analyzed by IC. A Dionex CarboPac PA-100 column (4 × 250 mm) was used to separate the oligosaccharides. The mobile phases were 100 mmol/L NaOH and 150 mmol/L NaAc, and the flow rate was 0.25 mL/min. The injection volume was 25 μL. The commercial neoagaro-oligosaccharides, including NA2, NA4, NA6, and NA8, were used as standards for TLC and IC analyses.

### 4.5. Fermentation Optimization of rAgaW1540 Production in E. coli Cells

To enhance product yields, fermentation optimization was performed to improve the expression condition of rAgaW1540 in *E. coli* cells. First, the IPTG concentration was optimized. Different concentrations (0, 0.1, 0.2, 0.4, 0.6, and 1.0 mM) of IPTG solution were added into the fermentation broth to reduce the recombinant proteins. Then, the agarose activity was measured by the method in section “Characterization of enzymatic properties”. The induction time was future optimized by determining the agarase activity in the fermentation broth after 4, 8, 12, 16, 20, and 24 h inductions by IPTG. The induction temperatures, including 12, 16, 20, 24, and 28 h, were also optimized to increase the production of rAgaW1540.

## Data Availability

The nucleotide sequence of the gene agaW1540 had been deposited in the GenBank database under the accession number MZ398234.
